# The Potassium Channel KCa3.1 Represents a Valid Pharmacological Target for Astrogliosis-Induced Neuronal Impairment in a Mouse Model of Alzheimer’s Disease

**DOI:** 10.3389/fphar.2016.00528

**Published:** 2017-01-05

**Authors:** Tianjiao Wei, Mengni Yi, Wen Gu, Lina Hou, Qin Lu, Zhihua Yu, Hongzhuan Chen

**Affiliations:** ^1^Department of Pharmacology, Institute of Medical Sciences, Shanghai Jiao Tong University School of MedicineShanghai, China; ^2^Department of Respiratory Medicine, Xinhua Hospital, Shanghai Jiao Tong University School of MedicineShanghai, China

**Keywords:** astrogliosis, Alzheimer’s disease, GFAP, inflammation, neurons

## Abstract

Alzheimer’s disease (AD) is a neurodegenerative disorder characterized by progressive decline of cognitive function. Astrogliosis plays a critical role in AD by instigating neuroinflammation, which leads ultimately to cognition decline. We previously showed that the intermediate-conductance Ca^2+^-activated potassium channel (KCa3.1) is involved in astrogliosis-induced by TGF-β *in vitro*. In the present study, we investigated the contribution of KCa3.1 channels to astrogliosis-mediated neuroinflammation, using Tg^APP/PS1^ mice as a model for AD. We found that KCa3.1 expression was increased in reactive astrocytes as well as in neurons in the brains of both Tg^APP/PS1^ mice and AD patients. Pharmacological blockade of KCa3.1 significantly reduced astrogliosis, microglial activation, neuronal loss, and memory deficits. KCa3.1 blockade inhibited astrocyte activation and reduced brain levels of IL-1β, TNF-α, iNOS, and COX-2. Furthermore, we used primary co-cultures of cortical neurons and astrocytes to demonstrate an important role for KCa3.1 in the process of astrogliosis-induced neuroinflammatory responses during amyloid-β (Aβ)-induced neuronal loss. KCa3.1 was found to be involved in the Aβ-induced activated biochemical profile of reactive astrocytes, which included activation of JNK MAPK and production of reactive oxygen species. Pharmacological blockade of KCa3.1 attenuated Aβ-induced reactive astrocytes and indirect, astrogliosis-mediated damage to neurons. Our data clearly indicate a role for astrogliosis in AD pathogenesis and suggest that KCa3.1 inhibition might represent a good therapeutic target for the treatment of AD.

**Highlights**:

(1) Blockade of KCa3.1 in APP/PS1 transgenic mice attenuated astrogliosis and neuron loss, and an attenuation of memory deficits. (2) Blockade of KCa3.1 attenuated Aβ-induced indirect, astrogliosis-mediated damage to neurons *in vitro* via activation of JNK and ROS.

## Introduction

Alzheimer’s disease (AD) is a neurodegenerative disorder characterized by progressive decline of cognitive function. The proposed mechanisms of cognitive impairment include synaptic dysfunction triggered by β-amyloid (Aβ), neuronal death, oxidative stress, tau pathology, and glutamate excitotoxicity. It is widely acknowledged that reactive gliosis plays a significant role in the progression of AD. Accumulation of reactive astrocytes and activation of microglia in affected brain regions are apparent in both AD patients and the majority of transgenic rodent models of AD. Garwood et al. (2011) reported that reactive astrogliosis is involved in regulating Aβ-induced neurotoxicity and tau phosphorylation. Reactive astrocytes release a variety of cytokines and pro-inflammatory mediators, which activate intracellular signaling pathways including extracellular signaling-related kinase (ERK), c-Jun N-terminal kinase (JNK), protein kinase C and PI3 kinase (Matos et al., 2008).

It has been reported that brief (5–15 min) application of Aβ to mixed disassociated hippocampal cultures evoked Ca^2+^ influx and up-regulated reactive oxygen species (ROS) in astrocytes, but not in neurons, whereas longer (24 h) exposure led to cell death, especially in neurons (Abramov et al., 2003). Although these data indicate that the primary target of Aβ might be astrocytes and that Aβ-induced oxidative stress and calcium overload in astrocytes may lead to neuronal death, the mechanism of these effects is not well understood.

In immune cells, airway smooth muscle cells and fibroblasts, the intermediate-conductance Ca^2+^-activated potassium channel KCa3.1 regulates membrane potential through K^+^ eﬄux, thus facilitating Ca^2+^ entry (Toyama et al., 2008; Di et al., 2010; Yu et al., 2013; Yi et al., 2016a). In the central nervous system (CNS), KCa3.1 plays a role in neuroprotection following traumatic brain injury, stroke and spinal cord injury (Bouhy et al., 2011). Up-regulation of KCa3.1 has been detected in reactive astrocytes in a mouse model of spinal cord injury. We have previously reported that blockade of KCa3.1, or gene deletion, attenuated TGF-β-induced astrogliosis by regulating intracellular Ca^2+^ in primary astrocytes (Yu et al., 2014). Yi et al. (2016a) reported that KCa3.1 is involved in scratch-induced migration of reactive astrocytes mediated by the JNK/c-Jun pathway. More recently, we demonstrated that blockade of KCa3.1 in senescence-accelerated mouse prone 8 (SAMP8) mice resulted in a decrease in astrogliosis and, moreover, an attenuation of memory deficits (Yi et al., 2016b). Evidence was found for astrogliosis in AD patients with mild cognitive impairment in a positron emission tomography study (Carter et al., 2012). Most recently, Schöll et al. (2015) reported that measure of astrogliosis in autosomal dominant AD could be observed decades before symptom onset using multi-tracer positron emission tomography, possibly coinciding with early fibrillar Aβ plaque deposition. Although reactive astrogliosis has long been recognized as a pathological feature of AD, the role of astrogliosis in the process of cognitive decline in AD is still poorly understood.

In the present study, we showed that blockade of KCa3.1 attenuated Aβ-induced changes in the biochemical profile of reactive astrocytes, including activation of JNK MAP kinase (MAPK) and production of ROS. An experimental KCa3.1 blocker, TRAM-34, attenuated Aβ-induced, indirect, astrogliosis-mediated damage to neurons *in vitro*, decreased astrogliosis and neuronal loss, and attenuated memory deficits in APP^Swe^/PS1^A246E^ transgenic (Tg^APP/PS1^) mice *in vivo.*

## Materials and Methods

### Brain Autopsy Material

Human brain sections were collected from six AD cases and six control cases (Netherlands Institute for Neuroscience, Amsterdam, Netherlands). Written informed consent for a brain autopsy to be used for research purposes after death had been obtained by the Netherlands Brain Bank.

### Animals

Nine-month male transgenic mice (the Jackson Lab, no. 003378, Tg^APP/PS1^) were purchased from the Jackson lab. Mice were divided into the following three groups: littermate wild type (WT) mice (*n* = 20), transgenic (Tg) mice with vehicle treatment (*n* = 20) or TRAM-34 (120 mg/kg, intraperitoneal, Apptec, Wuxi, China) treatment (Tg + TRAM-34, *n* = 20). After 4 weeks of drug treatment, the mice were submitted to behavioral testing, and then the mice were euthanized and brain tissues were collected for Western blotting or histology analyses. The protocol of animal experiments was approved by the Animal Experimentation Ethics Committee of Shanghai Jiao Tong University School of Medicine.

### Morris Water Maze Test

A modified Morris water maze test was carried out as previously described (Morris, 1984). The test requires the animals to find a visible or hidden platform in a large pool of opaque water and involves 5 days of training sessions (1 day with the platform visible and 4 days with the platform hidden), followed by a spatial probe trial, with no platform present, on day 6. During the hidden platform training, the mice were able to swim freely for 60 s to find a platform 1 cm below the water surface. During the spatial probe trial, the directness of the route taken to the area where the platform was previously located, together with the percentage of total time spent in this quadrant of the pool, was recorded using a video tracking system (Jiliang Software Technology Co., Ltd., Shanghai, China).

### Open Field Test

The open field test was carried out as described previously (Wu et al., 2016). Briefly, the mouse was gently placed in the center of an open field chamber (40 cm × 40 cm × 40 cm) and was allowed to move freely for 5 min. The movement parameters of the mouse were monitored and analyzed via a video camera connected to a tracking system (Jiliang Software Technology Co., Ltd., Shanghai, China). After each test, the floor of the open field was cleaned with solution of 70% ethanol to hide animal clues. The ratios of distance and time in the center were measured.

### Immunostaining and Data Analysis

The mice were anesthetized with chloral hydrate and perfused with 4% paraformaldehyde. The brain tissues were collected and cryoprotected with 30% sucrose in 0.1 M phosphate buffered saline (PBS). Brain sections (12 μm thick) were blocked with 3% bovine serum albumin in 0.1 M PBS for 1 h at room temperature and were then incubated with the following primary antibodies: mouse anti-KCa3.1 (1:200, Santa Cruz), rabbit anti-GFAP (1:1000; DAKO, Glostrup, Denmark), rabbit anti-NeuN (1:100, Millipore), rabbit anti-Iba1 (1:500, Wako) at 4°C overnight. Brain sections were labeled with either Alexa Fluor 488- or 568-conjugated anti-mouse or rabbit IgG (1:1000, Invitrogen). A TCS SP8 confocal laser scanning microscope (Leica, Germany), equipped with an argon-ion laser source, was used to capture images using excitation wavelengths of 405, 488, and 568 nm for DAPI, Alexa 488 and Alexa 568, respectively. Using the same reference position for each brain slice, between three and five random 0.01 mm^2^ microscopic fields were selected for quantification. Quantification was carried out in six slices of each brain (120 μm intervals), using immunoreactivity for GFAP, Iba1, and NeuN expression. The numbers of GFAP^+^, Iba1^+^, and NeuN^+^ positive cells were counted in six slices per mouse in a blinded manner, using Leica LAS AF Lite software to measure the areas.

### Senile Plaque Staining

For senile plaques staining, coronal sections (12 μm) were cut using a Leica cryostat (Leica CM1850). Primary 6E10 antibody (SIG-39300, Covance, Princeton, NJ, USA) was used on the sections overnight at 4°C. Sections were incubated with biotinylated secondary antibody (AK-6602, Vector) for 45 min. Sections were developed using the ABC elite kit (AK-6600, Vector). Image-Pro plus software (Media cybernetics, USA) was used to measure and recorded as the average plaque areas per field. Six slices per mouse were used to count the plaque number in a blinded manner.

### Enzyme-Linked Immunosorbent Assay

ELISA was performed using the kit for TNF-α and IL-1β (Rapidbio Labs, Langka Trade Co. Ltd., Shanghai, China). The procedures were conducted according to manufacturer’s protocols.

### Preparation of Oligomeric Aβ_1-42_ Peptides

Our preparation of oligomeric amyloid β (Aβ1-42) follows the procedure described previously (Wang et al., 2012). As described previously, monomeric peptide Aβ1-42 was initially dissolved in 1,1,1,3,3,3-hexafluoro 2-propanol (HFIP, Sigma, St. Louis, MO, USA) at 1 mg/ml. Dried peptides were resolved in dimethyl sulfoxide (DMSO), diluted to 100 μM with ddH_2_O, and incubated at 4°C for 24 h.

### Primary Cultures

Primary cortical astrocyte cultures derived from neonatal (P0–P2) C57BL/6J mice were prepared from mixed glial cultures (10–14 days *in vitro*) as described previously (Wang et al., 2008). Astrocyte-conditioned medium (ACM) for treating neurons was obtained using the following procedure. Firstly, astrocytes were cultured in Dulbecco’s Modified Eagle’s medium (DMEM) contained 10% fetal bovine serum. The culture medium (CM) was changed to neurobasal medium with B27 supplement (Invitrogen) after confluent astrocytes were serum-free for 24 h. The NB/B27-based astrocytes were then treated with Aβ_1-42_ oligomer (5 μM) for different lengths of time. The CM from the NB/B27-based cells was collected and used immediately.

Neuronal cultures were established by adding cytosine arabinoside (2 μM) to the mixed glial cultures to inhibit proliferation of glial cells. The neurons were cultured for at least 14 days before treatment with astrocytes CM.

Primary mixed cortical cells were isolated from neonatal (P0–P2) C57BL/6J mice and cultured as described previously (Rodriguez-Gonzalez et al., 2016). Briefly, astrocytes were collected and seeded in multiwell plates in DMEM. Four days after plating, neurons were obtained from brain cortices of P0 C57BL/6J mice and seeded on top of the astrocyte layer.

A Cell Counting Kit-8 (CCK-8, Dojindo Laboratories, Kumamoto, Japan) was used to measure cell viability (Yu et al., 2013).

### Neurite Outgrowth Assay

Aβ_1-42_ oligomers were added to the primary cultured neurons with or without TRAM-34 and then stained with primary antibody microtubule associated protein 2 (MAP2) plus Alexa Fluor 555-conjugated secondary antibody and DAPI. Cellomics KineticScan reader was used to scan the MAP2 positive cells. Extended Neurite Outgrowth software (Thermo Scientific, Philadelphia, PA, USA) was used to analyze the data.

### Western Blot Analysis

Protein extracts were separated by 10 % (w/v) sodium dodecyl sulfate-polyacrylamide gel electrophoresis and were transferred to polyvinylidene difluoride membrane. The membrane was incubated with following primary antibodies for anti-total p38/JNK/ERK MAPK, anti-phospho-p38/JNK/ERK MAPK antibodies (1:1000, Cell Signaling Technology, Danvers, MA, USA), anti-Synaptophysin antibody (1:1000, Abcam), anti-PSD95 antibody (1:1000, Abcam), anti-NeuN antibody (1:1000, Millipore), anti-MAP2 antibody (1:1000, Abcam), anti-GFAP antibody (1:5000, Dako, Glostrup, Denmark), anti-KCa3.1 antibody (1:400, Abcam), and anti-β-actin antibody (1:3000, Sigma). Secondary antibodies were horseradish peroxidase-conjugated antibody (1:3000; Amersham Biosciences) for 1 h at room temperature.

### Reactive Oxygen Species Measurement

Aβ_1-42_ oligomers were added to the primary cultured astrocytes with or without TRAM-34. Astrocytes were loaded with 30 μM 5-(and-6)-chloromethyl–2′,7′-dichlorodihydrofluorescein diacetate (CM-H_2_DCFDA, Invitrogen, Waltham, CA, USA) to measure ROS generation as previously described (Alberdi et al., 2013).

### Statistical Analysis

Statistical significance was analyzed using the Student’s *t-*test, one-way ANOVA followed by Dunnett’s *post hoc* tests or two way ANOVA followed by Bonferroni *post hoc* test, depending on the case, using Prism software (GraphPad Software, Inc., La Jolla, CA, USA). Results are presented as Mean ± SEM, with *p* < 0.05 considered to be statistically significant.

## Results

### KCa3.1 Is Up-Regulated in Reactive Astrocytes of Tg^APP/PS1^ Mice and AD Patients

**FIGURE 1:**
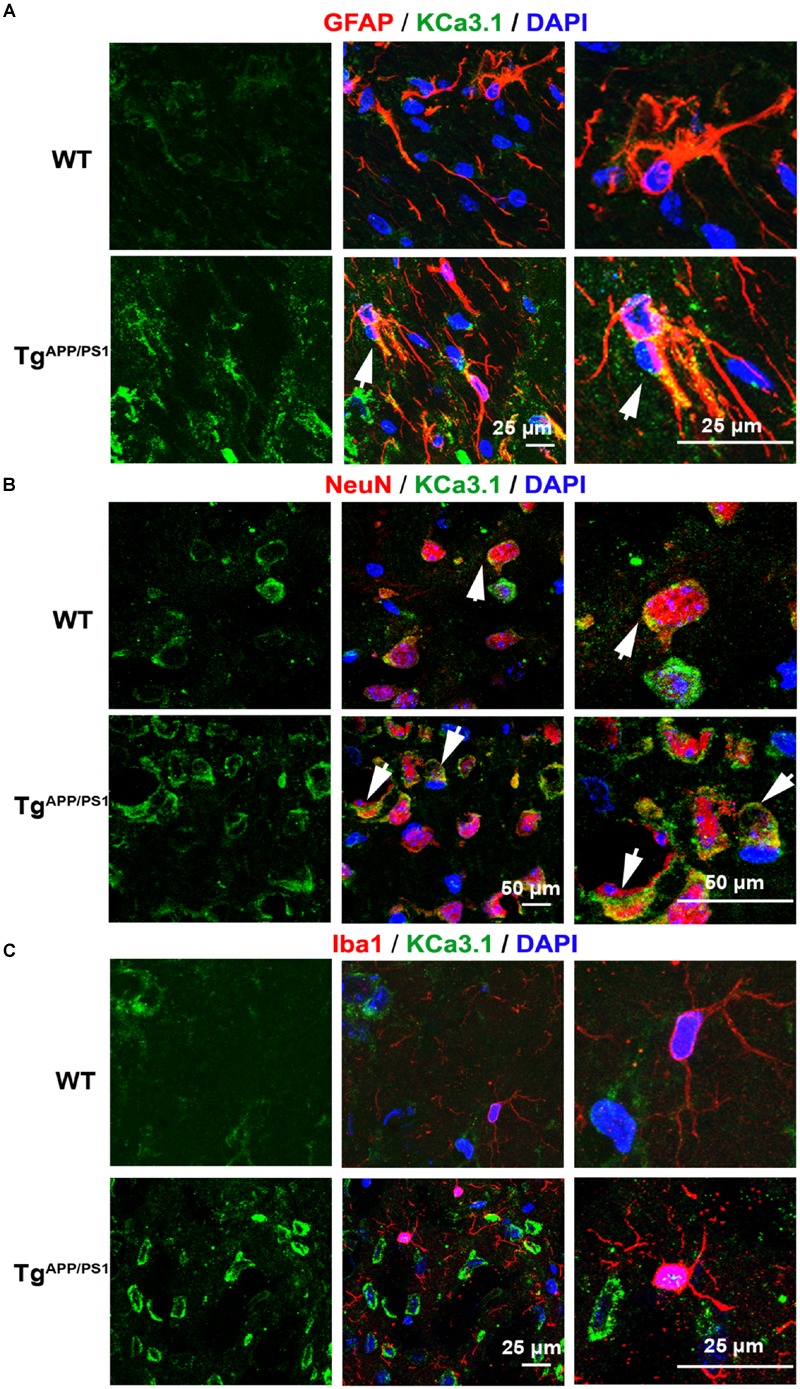
**Up-regulation of KCa3.1 channels in astrocytes and neurons in Tg^APP/PS1^ mouse brains.** Immunofluorescence double staining of KCa3.1 (green) with GFAP, NeuN, or Iba1 (red) in brain sections of 9 month old wild type (WT) and Tg^APP/PS1^ mice. Co-staining of **(A)** KCa3.1 and GFAP, **(B)** KCa3.1 and NeuN, and **(C)** KCa3.1 and Iba1 in WT and Tg^APP/PS1^ mice. Arrows indicate co-labeling of **(A)** KCa3.1 and GFAP, **(B)** KCa3.1 and NeuN, and (C) KCa3.1 and Iba1. Views are enlarged in the adjacent panels. Nuclei are stained blue with DAPI. Scale bars: **(A)** 25 μm, **(B)** 50 μm, and **(C)** 25 μm.

**FIGURE 2:**
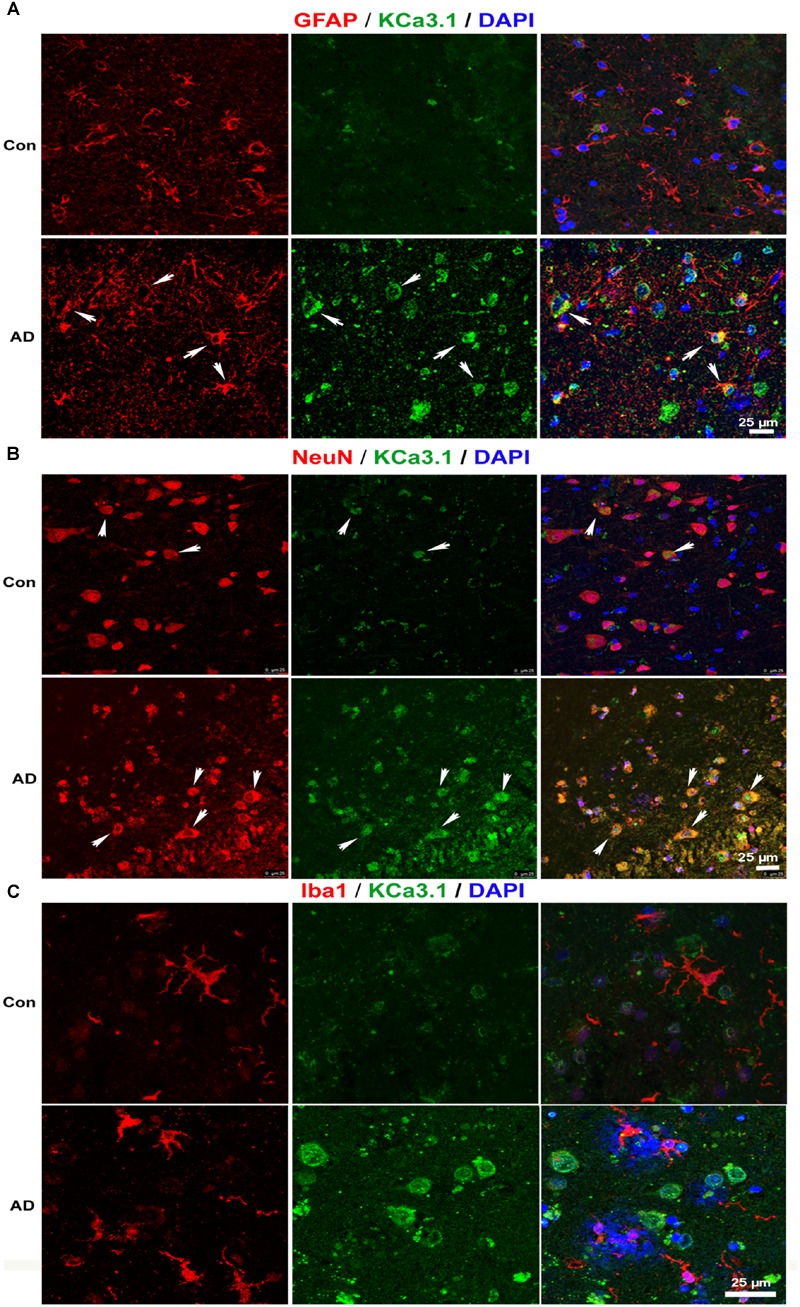
**Up-regulation of KCa3.1 channels in astrocytes and neurons in brains of Alzheimer’s disease (AD) patients.** Immunofluorescence double staining of KCa3.1 (green) with GFAP, NeuN, or Iba1 (red) in brain slices of healthy individuals and AD patients. Co-staining of **(A)** KCa3.1 and GFAP, **(B)** KCa3.1 and NeuN, and **(C)** KCa3.1 and Iba1 in healthy individuals and AD patients. Arrows indicate double staining of **(A)** KCa3.1 and GFAP, **(B)** KCa3.1 and NeuN, and **(C)** KCa3.1 and Iba1. Nuclei are stained blue with DAPI. Scale bar: 25 μm.

We recently showed that KCa3.1 expression is increased in both reactive astrocytes and neurons in the brains of SAMP8 mice, a model that is generally used to investigate the mechanisms of age-related memory deficits (Yi et al., 2016b). In the present study, expression of KCa3.1 was detected in the brains of Tg^APP/PS1^ mice at 9 months of age and in AD patients. Age-matched WT littermates and healthy humans were used as controls. In WT mice (**Figure 1A**) and healthy humans (**Figure 2A**), there was little co-localization between KCa3.1 and GFAP^+^ astrocytes. In 9 month old Tg^APP/PS1^ mice (**Figure 1A**) and AD patients (**Figure 2A**), KCa3.1 was detected in GFAP^+^ hypertrophic astrogliosis. Kaushal et al. (2007) reported that KCa3.1 is expressed in rat neurons *in vitro*. In the present study, expression of KCa3.1 was shown to be higher in NeuN^+^ neurons of Tg^APP/PS1^ mice (**Figure 1B**) and AD patients (**Figure 2B**) than in WT mice and control humans, where expression levels are low. Although there is convincing evidence for the expression of KCa3.1 on microglia *in vitro*, data supporting expression of KCa3.1 on microglia *in vivo* are scarce (Kaushal et al., 2007). In this study, rare co-localization of KCa3.1 and Iba1^+^ microglia was detected in brain slices of both WT and Tg^APP/PS1^ mice (**Figure 1C**) and control humans and AD patients (**Figure 2C**).

KCa3.1 was expressed at low levels, mainly in neurons, in WT mice and control humans. In Tg^APP/PS1^ mice and AD patients, KCa3.1 expression was up-regulated in neurons and was also observed in astrocytes.

### Blockade of KCa3.1 Rescues Memory Deficits and Spontaneous Motor Activity in Tg^APP/PS1^ Mice

**FIGURE 3:**
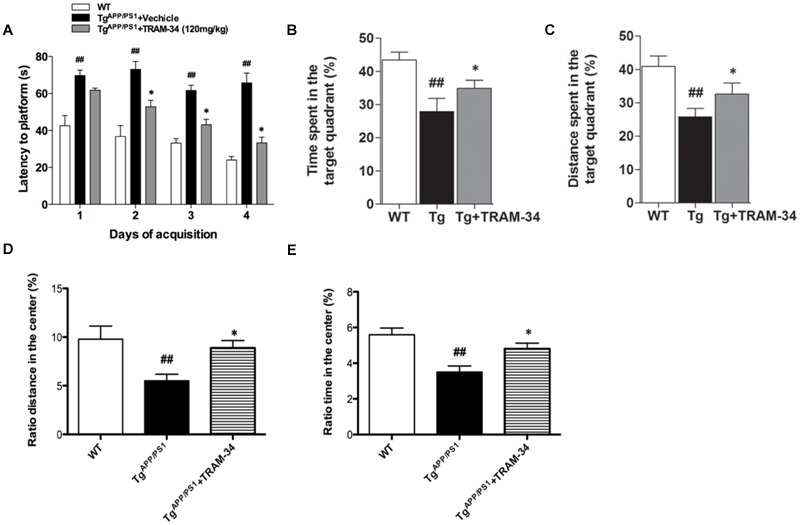
**Blockade of KCa3.1 rescued memory deficits and spontaneous motor activity in Tg^APP/PS1^ mice.** The Morris water maze test **(A–C)** and open field test **(D,E)** were performed on 9 months old mice as described in the section “Materials and Methods.” Tg^APP/PS1^ mice were treated with vehicle or TRAM-34 (120 mg/kg, intraperitoneally) daily for 4 weeks. **(A)** Average latency to hidden platform, **(B)** Percentage of time spent by each group swimming in target quadrant during probe trial (no platform), **(C)** Percentage of total swim distance by each group in target quadrant during probe trial (no platform), **(D,E)** Exploratory locomotion of the open field test, **(D)** Ratio of distance in exploratory locomotion of the open field test, and **(E)** Ratio of time in center in exploratory locomotion of the open field test. Tg^APP/PS1^ group showed decreased locomotion compared with Tg^APP/PS1^ + TRAM-34 (120 mg/kg, intraperitoneally) group. Data represent mean ± SEM (*n* = 20 per group). ^##^*p* < 0.01 versus WT mice. ^∗^*p* < 0.05 versus vehicle-treated Tg^APP/PS1^ mice. Tg, Tg^APP/PS1^.

In a previous study, we showed that inhibition of KCa3.1 channels attenuated memory deficits in SAMP8 mice (Yi et al., 2016b). In the present study, TRAM-34 (120 mg/kg, intraperitoneally) was used to test whether pharmacological blockade of KCa3.1 would attenuate loss of memory and spontaneous motor activity in Tg^APP/PS1^ mice, and the dosage of TRAM-34 was used as previously described (Bouhy et al., 2011; Chen et al., 2016; Yi et al., 2016b). Nine months old Tg^APP/PS1^ mice were treated once daily with TRAM-34 (120 mg/kg, intraperitoneally) or vehicle for 4 weeks and the Morris water maze test and open field test were then used to assess memory and motor activity, respectively. In the hidden platform arm of the Morris water maze test, the Tg^APP/PS1^ + vehicle group showed memory deficits compared with WT mice. The Tg^APP/PS1^ + TRAM-34 group showed significantly improved spatial learning and memory compared with the Tg^APP/PS1^ + vehicle group (*p* < 0.05, **Figure 3A**). In the spatial probe trial without an escape platform, the Tg^APP/PS1^ + TRAM-34 group spent more time and swam for greater distances in the target quadrant than the Tg^APP/PS1^ + vehicle group (*p* < 0.05, **Figures 3B,C**).

In the open field test, the Tg^APP/PS1^ + vehicle group had a decreased ratio distance (*p* < 0.01, **Figure 3D**) and ratio time (*p* < 0.01, **Figure 3E**) in the center compared to WT mice. In contrast, the Tg^APP/PS1^ + TRAM-34 group had a significantly greater ratio distance (*p* < 0.05, **Figure 3D**) and more ratio time (*p* < 0.05, **Figure 3E**) in the center than the Tg^APP/PS1^ + vehicle group.

### Blockade of KCa3.1 Attenuates Gliosis and Loss of Neurons in Tg^APP/PS1^ Mice

**FIGURE 4:**
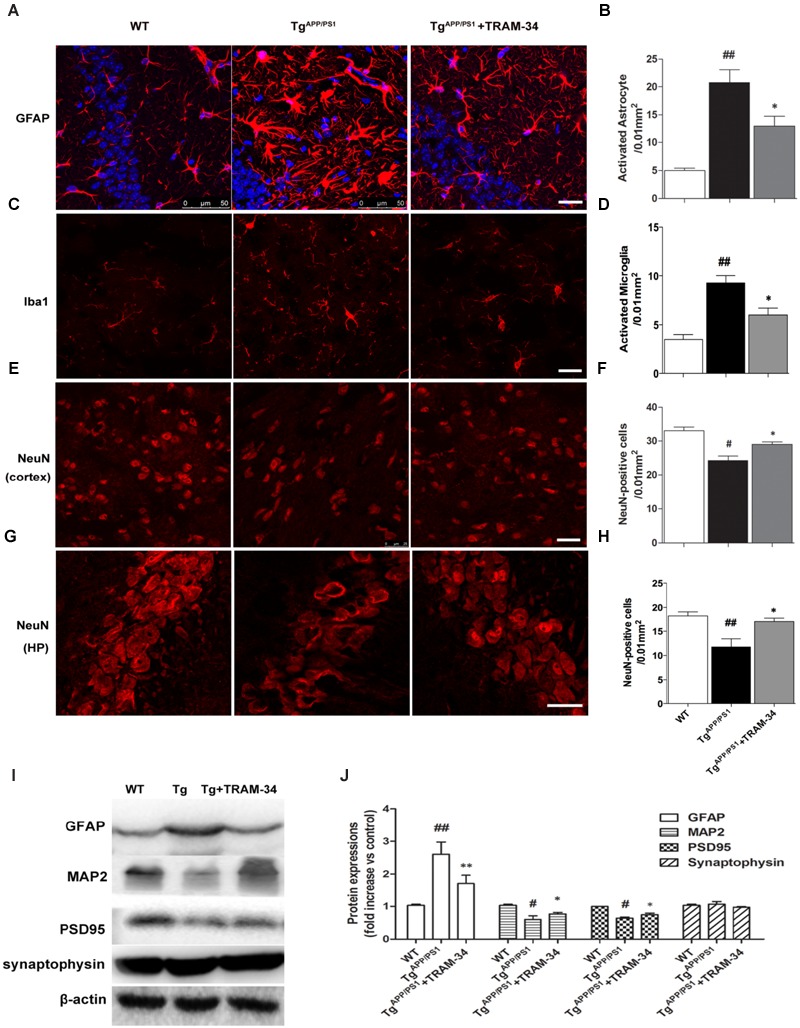
**Blockade of KCa3.1 attenuated gliosis and neuronal loss in Tg^APP/PS1^ mice. (A)** Representative images of GFAP-immunoreactive astrocytes from hippocampal regions of WT and Tg mice, with and without TRAM-34 (120 mg/kg/d). **(B)** Quantification of reactive astrocyte number/0.01 mm^2^ in hippocampus (*n* = 6–8). **(C)** Iba1 immunoreactivity of activated microglia from hippocampal regions of WT and Tg mice, with and without TRAM-34 (120 mg/kg/d). **(D)** Quantification of activated microglial number/0.01 mm^2^ in hippocampus (*n* = 6–8). NeuN immunoreactivity of neurons in **(E)** cortex and **(G)** hippocampal regions of WT and Tg mice, with and without TRAM-34 (120 mg/kg/d). Quantification of neuron number/0.01 mm^2^ in **(F)** cortex and **(H)** hippocampus (*n* = 6–8). Data represent mean ± SEM. ^#^*p* < 0.05, ^##^*p* < 0.01 compared with WT mice. ^∗^*p* < 0.05, versus Tg mice (*n* = 6–8). **(I)** Western blot analysis using antibodies to astrocyte marker GFAP, dendritic protein MAP2, post-synaptic protein PSD95, and pre-synaptic protein synaptophysin. **(J)** Results are presented as mean ± SEM (*n* = 4). ^#^*p* < 0.05, ^##^*p* < 0.01 compared with WT mice. ^∗^*p* < 0.05, ^∗∗^*p* < 0.01 compared with Tg mice. Tg, Tg^APP/PS1^. Scale bar: 25 μm.

Reactive astrogliosis is a common pathological feature of chronic neurological diseases associated with aging, such as AD (Khakh and Sofroniew, 2015). We have previously shown that inhibition of KCa3.1 reduces the reactive astrogliosis response (Yu et al., 2014). In the present study, both GFAP^+^-reactive astrocytes and Iba1^+^-activated microglia were significantly increased in the Tg^APP/PS1^ + vehicle group compared with WT mice. The Tg^APP/PS1^ + TRAM-34 group, however, showed a significantly suppressed astrogliosis response (*p* < 0.05, **Figures 4A,B**) and reduced numbers of activated microglia (*p* < 0.05, **Figures 4C,D**) compared with the Tg^APP/PS1^ + vehicle group. The Tg^APP/PS1^ + TRAM-34 group also showed reduced neuronal loss in the cortex (*p* < 0.05, **Figures 4E,F**) and CA1 hippocampus (*p* < 0.05, **Figures 4G,H**). Significantly more NeuN^+^ neurons were present in the Tg^APP/PS1^ + TRAM-34 group than in the Tg^APP/PS1^ + vehicle group.

Because dendritic and synaptic damage, as well as neuronal loss, likely play important roles in the process of AD, we questioned whether astrogliosis damages dendrites and synapses. Western blotting demonstrated that treatment with TRAM-34 (120 mg/kg) significantly reduced levels of astrogliosis and increased expression of dendritic marker MAP2 and excitatory synapses marker PSD95, but not the pre-synaptic marker synaptophysin, in Tg^APP/PS1^ mice (**Figures 4I,J**).

### Blockade of KCa3.1 Decreases Amyloid Deposition and Inflammatory Cytokine Production in Tg^APP/PS1^ Mice

**FIGURE 5:**
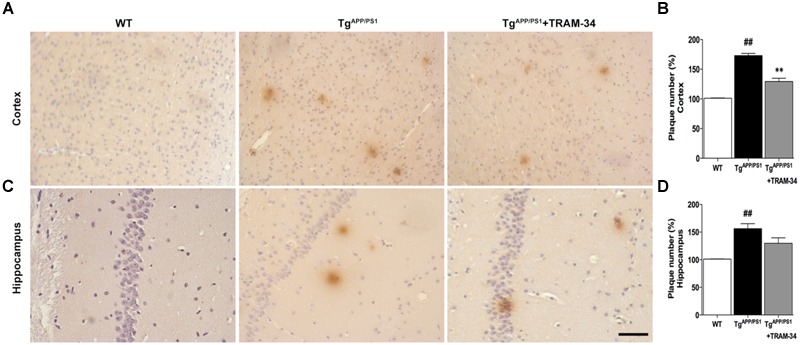
**Blockade of KCa3.1 attenuated β-amyloid burden in the brains of Tg^APP/PS1^ mice.** Mouse anti-6E10 monoclonal antibody was used to detect senile plaques in **(A)** brain cortex and **(C)** hippocampus of WT, Tg^APP/PS1^, and Tg^APP/PS1^ + TRAM-34 (120 mg/kg) groups (*n* = 10 per group). Quantification of senile plaque numbers in **(B)** cortex and **(D)** hippocampus. Data represent mean ± SEM. ^##^*p* < 0.01, versus WT mice. ^∗∗^*p* < 0.01, versus vehicle-treated Tg^APP/PS1^ mice. Scale bar: 50 μm.

To investigate whether inhibition of KCa3.1 channels can attenuate amyloid accumulation, cerebral senile plaques in the different groups of mice were visualized using 6E10 staining. Senile plaque formation in both cortex and hippocampus was significantly increased in the Tg^APP/PS1^ + vehicle group compared with WT mice (*p* < 0.01, **Figures 5A–D**). After treatment with TRAM-34 (120 mg/kg, i.p.) for 4 weeks, Tg^APP/PS1^ mice showed decreased senile plaque formation. Quantification showed that senile plaque formation was decreased in the cortex (*p* < 0.01; **Figure 5B**) but not in the hippocampus (*p* > 0.05; **Figure 5D**) in the Tg^APP/PS1^ + TRAM-34 group compared with the Tg^APP/PS1^ + vehicle group. Given the contribution of gliosis to Aβ production, the decrease in senile plaque formation seen in the cortex of Tg^APP/PS1^ + TRAM-34 group is likely attributable to a reduction in numbers of reactive astrocytes and active microglia, brought about by inhibition of KCa3.1 channels.

**FIGURE 6:**
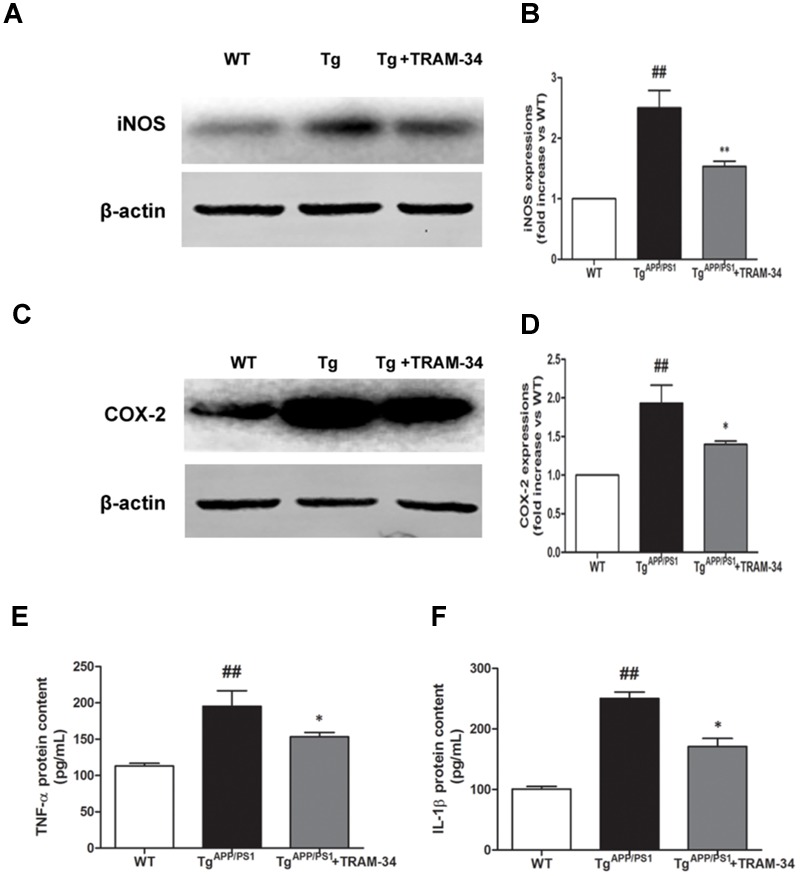
**Blockade of KCa3.1 attenuated expression and release of inflammatory mediators in the brains of Tg^APP/PS1^ mice.** Western blots showing protein expression of **(A,B)** iNOS and **(C,D)** COX-2 proteins in the brains of WT and Tg^APP/PS1^ mice, with or without treatment with TRAM-34 (*n* = 4), at the age of 9 months. Measurement of **(E)** TNF-α and **(F)** IL-1β released by ELISA in homogenated cortex of WT and Tg^APP/PS1^ mice, with or without treatment with TRAM-34 (*n* = 4) at the age of 9 months. Data represent mean ± SEM. ^##^*p* < 0.01 compared with WT mice. ^∗^*p* < 0.05, ^∗∗^*p* < 0.01 compared with Tg^APP/PS1^ mice.

Neuronal death in AD is largely caused by the release of pro-inflammatory mediators during gliosis (Scuderi et al., 2014). Blockade of KCa3.1 inhibited the up-regulation of iNOS (**Figures 6A,B**) and COX-2 (**Figures 6C,D**), compared with vehicle-treated Tg^APP/PS1^ mice. Levels of IL-1β and TNF-α in brain homogenates from the different groups were measured by ELISA experiments (**Figures 6E,F**). Levels of both IL-1β and TNF-α released were attenuated in the Tg^APP/PS1^ + TRAM-34 group compared with the Tg^APP/PS1^ + vehicle group. These data demonstrate that KCa3.1 is involved in activation of glial cells in a mouse model of AD.

### Blockade of KCa3.1 Attenuated Aβ-Induced Reactive Astrogliosis through JNK Signaling Pathways

**FIGURE 7:**
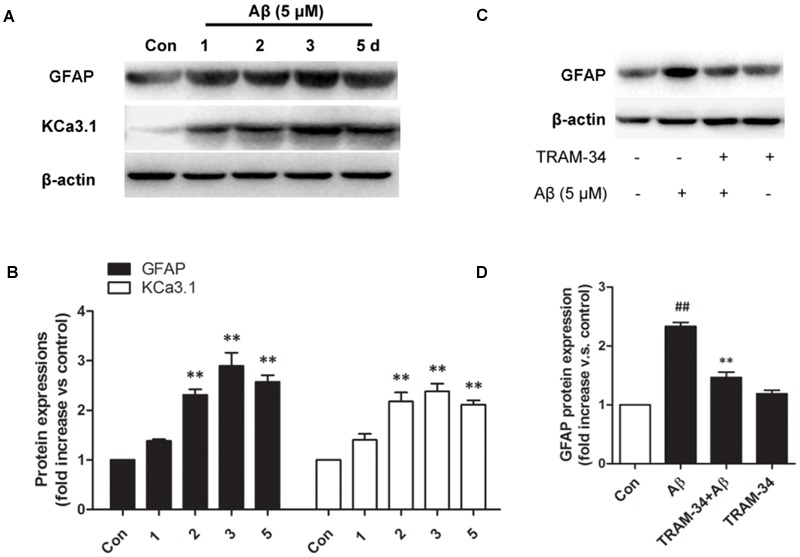
**KCa3.1 is involved in Aβ oligomer-induced astrogliosis. (A)** Western blot showing protein expression of astrocytic GFAP and KCa3.1 after treatment with Aβ (5 μM) for 1, 2, 3, or 5 days (*n* = 3). **(B)** Data are presented as mean ± SEM. ^∗∗^*p* < 0.01 compared with control. **(C)** Western blot showing expression of GFAP protein after stimulation with Aβ (5 μM), with or without TRAM-34 (1 μM) for 3 days (*n* = 3). **(D)** Data represent mean ± SEM. ^∗∗^*p* < 0.01 compared with Aβ-treatment group; ^##^*p* < 0.01 compared with control. Con, control.

We first tested whether Aβ-induced astrogliosis was associated with KCa3.1 expression *in vitro*. Primary astrocytes cultured in the presence of Aβ (5 μM) showed up-regulated GFAP and KCa3.1 (**Figures 7A,B**). The up-regulation of GFAP was blocked by pre-treatment with TRAM-34 (1 μM; **Figures 7C,D**). The concentration of TRAM-34 (1 μM) was used as previously described (Bouhy et al., 2011; Yu et al., 2014).

**FIGURE 8:**
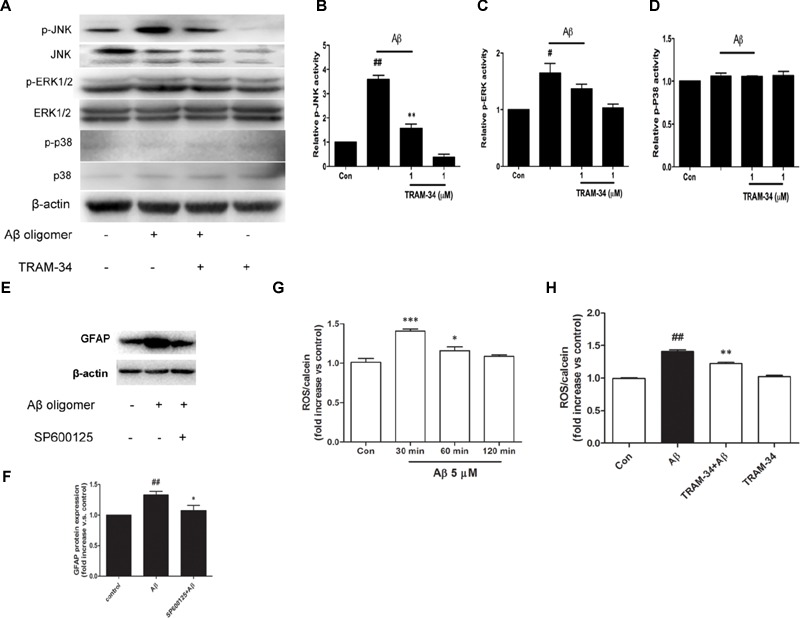
**KCa3.1 is involved in Aβ-induced generation of activated astrocyte phenotype. (A)** Representative images of total JNK/ERK/P38 and p-JNK/p-ERK/p-P38, in control astrocytes and Aβ-stimulated astrocytes (30 min), with or without TRAM-34 (1 μM) treatment. **(B–D)** Mean values of p-JNK/p-ERK/p-P38 activity relative to total JNK/ERK/P38 (*n* = 3–6). Results are presented as mean ± SEM. ^#^*p* < 0.05, ^##^*p* < 0.01 versus control, ^∗∗^*p* < 0.01 versus Aβ-stimulated alone. **(E,F)** Western blots showing expression of GFAP protein in astrocytes after 3 days Aβ stimulation in the presence of SP600125 (10 μM; *n* = 3). Data are presented as mean ± SEM. ^##^*p* < 0.01 versus control, ^∗^*p* < 0.05 versus Aβ. **(G)** CM-H2DCFDA (30 μM) was used to measure the level of reactive oxygen species (ROS) generation induced by Aβ in astrocytes. **(H)** ROS generation was reduced by TRAM-34 (1 μM) in astrocytes treated with Aβ (*n* = 5). Data represent mean ± SEM. β-actin was used as an internal control. ^##^*p* < 0.01, ^∗∗∗^*p* < 0.001, ^∗^*p* < 0.05 compared with control; ^∗∗^*p* < 0.01 compared with Aβ-treated cells.

Reactive astrocytes release a variety of cytokines and pro-inflammatory mediators, which activate intracellular MAPK signaling pathways (Matos et al., 2008). We examined whether the involvement of KCa3.1 in Aβ-induced astrogliosis is mediated by the ERK, JNK, and p38 signaling pathways (**Figures 8A–D**). Astrocytes exposed to Aβ (5 μM) for 30 min showed up-regulated phosphorylation of JNK (**Figures 8A,B**) and ERK (**Figures 8A,C**) but only phosphorylation of JNK was inhibited by TRAM-34 (1 μM; **Figure 8B**). Treatment with the JNK inhibitor SP600125 (10 μM) for 3 days attenuated Aβ-induced GFAP protein expression in astrocytes (**Figures 8E,F**).

The molecular probe CM-H_2_DCFDA, which detects intracellular ROS levels, was used to determine whether KCa3.1 contributed to ROS production *via* Aβ-induced astrogliosis. After treatment with Aβ oligomer (5 μM) for 30 min, a rapid transient increase in the DCF signal was detected (**Figure 8G**). ROS production at 30 min was inhibited by TRAM-34 (*p* < 0.01, **Figure 8H**). These data demonstrate that KCa3.1 is involved in reactive astrocytosis and oxidative stress *via* the JNK signaling cascades.

### Blockade of KCa3.1 Attenuated Indirect Aβ-Induced Neurotoxicity Mediated by Astrocytes

**FIGURE 9:**
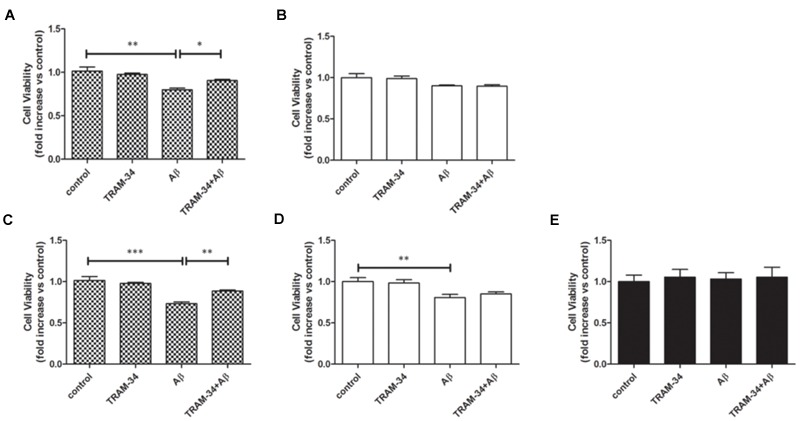
**KCa3.1 is involved in astrocyte-accelerated Aβ-induced neurotoxicity.** Bar charts showing cell viability of **(A,C)** mouse primary mixed cultures and **(B,D)** neuronal cultures treated with Aβ (5 μM) for **(A,B)** 48 or **(C,D)** 72 h, with or without pre-treatment with TRAM-34 (1 μM). **(E)** Cell viability of mouse primary astrocytes treated for 72 h with Aβ (5 μM), with and without pre-treatment with TRAM-34 (1 μM). Data represent mean ± SEM of cell viability relative to control (*n* = 4 cultures). ^∗^*p* < 0.05, ^∗∗^*p* < 0.01, ^∗∗∗^*p* < 0.001.

It has been reported that astrocytes accelerate Aβ-induced neurotoxicity (Garwood et al., 2011). To determine whether or not KCa3.1 is involved in astrocyte-accelerated Aβ-induced neurotoxicity, neurons, astrocytes and mixed cultures (neurons with astrocytes) were treated with 5 μM Aβ oligomers and then cell viability was measured in the different groups at 48 or 72 h (**Figures 9A–E**). Treatment with 1 μM TRAM-34 alone did not significantly change the viability of any of these cell cultures. Pre-treatment of mixed cultures, but not neuronal cultures, with TRAM-34 before treatment with Aβ increased cell viability at both 48 and 72 h (**Figures 9A,C**), suggesting that TRAM-34 might reduce Aβ-induced neurotoxicity by suppressing astrogliosis. A significant decrease in cell viability was observed only after 72 h in Aβ-treated neuronal cultures (**Figures 9B,D**). Aβ oligomers did not cause decreased viability from astrocytes at 72 h (**Figure 9E**), indicating that the phenotype switch of astrogliosis significantly accelerates Aβ-induced neurotoxicity.

**FIGURE 10:**
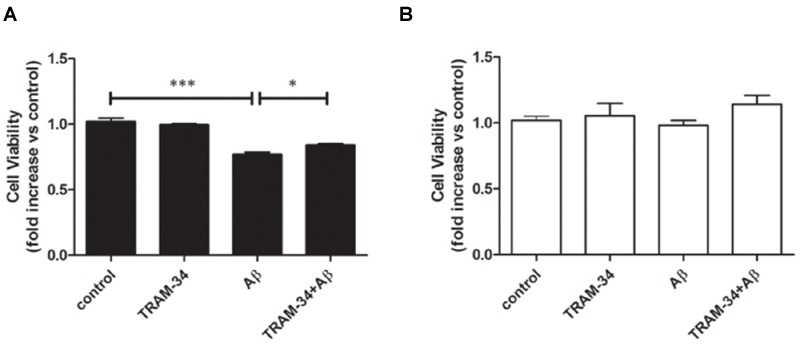
**Aβ-induced indirect, astrogliosis-mediated neurotoxicity.** Bar charts showed cell viability of neurons exposed to **(A)** CM from astrocytes treated with Aβ (5 μM), with or without TRAM-34 (1 μM) and **(B)** cell-free astrocytes culture medium treated with Aβ (5 μM) for 48 h, with or without TRAM-34 (1 μM). Data represent mean ± SEM of cell viability relative to control (*n* = 4 cultures). ^∗^*p* < 0.05, ^∗∗∗^*p* < 0.001. CM, conditioned medium.

We next asked whether blockade of KCa3.1 might attenuate neurotoxicity induced by inflammatory mediators during astrogliosis. Pre-treatment with TRAM-34 (1 μM) was found to rescue neuronal viability in cultures treated with astrocytes CM (**Figure 10A**). As a negative control, neuronal viability was not significantly affected by cell-free astrocyte CM that had been incubated for 48 h with Aβ (5 μM; **Figure 10B**).

**FIGURE 11:**
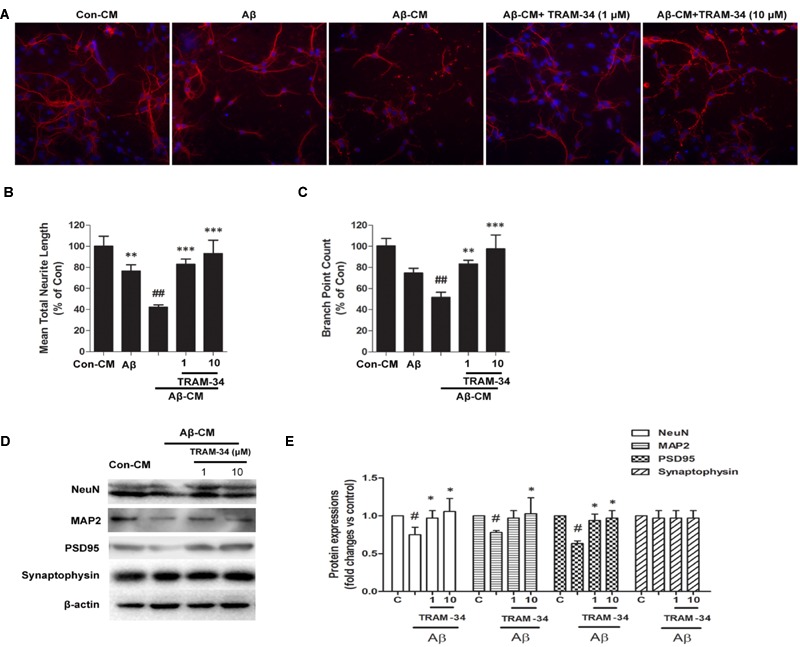
**Blockade of KCa3.1 attenuated Aβ-induced, indirect, astrocyte-mediated damage to dendrites and synapses.** Levels of dendritic and synaptic markers were compared between hippocampal neurons treated with solvent only (control treatment), 5 μM Aβ, Aβ-CM, or CM from astrocyte cultures in which Aβ-induced activation was inhibited for 24 h by TRAM-34 (TRAM-34 + Aβ-CM). A Cellomics KineticScan HCS Reader was used to image the primary cerebral neurons. **(A)** For better visualization of individual dendrites, dendrites of the sparsely plated neurons were immunostained with MAP2 (red), and nuclei were stained with DAPI (blue). Extended Neurite Outgrowth bioapplication software was used to analyze **(B)** neurite length and **(C)** branch point counts. Data represent mean ± SEM (*n* = 3). ^∗∗^*p* < 0.01, ^∗∗∗^*p* < 0.001 compared with Aβ-CM. ^##^*p* < 0.01 compared with control. Aβ-CM treatment significantly reduced neurite length and branch point counts. This reduction was prevented by inhibition of astrocyte activation by TRAM-34 (1 and 10 μM; *n* = 3). **(D)** Western blot analysis with the following primary antibodies: neuronal marker NeuN, dendritic marker MAP2, post-synaptic proteins PSD95, and pre-synaptic protein synaptophysin. **(E)** Results are presented as mean ± SEM (*n* = 3). ^#^*p* < 0.05 compared with con-CM. ^∗^*p* < 0.05 compared with Aβ-CM. CM, conditioned medium.

Because damage to dendrites and synapses occurs before neuronal loss during the development of AD (Szegedi et al., 2006), we asked whether Aβ-induced astrogliosis-mediated by KCa3.1 could accelerate damage to dendrites and synapses. More signs of dendritic and synaptic damage were observed in neurons treated with Aβ-treated astrocytes CM than in those treated with control CM, as demonstrated by immunofluorescent staining for the dendritic marker MAP2 (**Figure 11A**). Incubation with Aβ-CM decreased total neurite length (**Figure 11B**) and number of branch points (**Figure 11C**). Pre-incubation with TRAM-34 (1 and 10 μM) markedly reversed the effect of Aβ-CM by increasing total neurite length and number of branch points (**Figures 11A–C**). The concentrations of TRAM-34 (1 and 10 μM) were used as previously described (Lallet-Daher et al., 2009; Yu et al., 2014). Western blots showed that blockade of KCa3.1 attenuated Aβ-CM-induced decreases in NeuN, MAP2, and PSD95, but not decreases in the pre-synaptic marker, synaptophysin (**Figures 11D,E**). Taken together, these results show that KCa3.1 is involved in Aβ-mediated damage to dendrites and synapses by an indirect, astrogliosis-mediated mechanism.

## Discussion

The data presented herein demonstrate that blockade of KCa3.1 attenuates neuropathology by regulating neuroinflammation in a mouse model of AD and, moreover, that prevention of astrogliosis might be a promising strategy for the treatment of AD. Using Tg^APP/PS1^ mice as a model of AD, we have shown that pharmacological blockade of KCa3.1 significantly reduced astrogliosis, neuronal loss, and memory deficits. KCa3.1 blockade inhibited astrocyte activation and reduced levels of IL-1β, TNF-α, iNOS, and COX-2 in the brain. *In vitro* experiments with primary murine neuronal, astrocytic and mixed cortical cultures exposed to Aβ confirmed that blockade of KCa3.1 improved neuronal survival by reducing astrocyte activation and ROS production.

Deposits of Aβ could induce oxidative stress, glial activation, and local cell loss. In the progression of AD, astrocytes undergo a switch to a reactive phenotype, characterized by profound functional and morphological alterations (O’Callaghan et al., 2014). Up-regulation of the astrogliosis marker GFAP is a well-characterized example of these phenotypical changes and, in our cellular model, TRAM-34 was found to negatively modulate expression of both GFAP and KCa3.1.

There is a large body of data that implicates MAPK signaling pathways, especially JNK pathways, in oxidative stress (Matos et al., 2008). In the present study, blockade of KCa3.1 attenuated phosphorylation of JNK, which is activated by Aβ stimulation. Astrogliosis has been shown to contribute to inflammatory processes in AD through the release of ROS, pro-inflammatory factors, and cytokines (Heneka et al., 2001; Verkhratsky et al., 2010). For example, IL-1β, TNF-α, ROS, and NO have been found to regulate pro- and anti-inflammatory genes, including NOS-2 and COX-2 (Tuppo and Arias, 2005; Wyss-Coray, 2006). We found that blockade of KCa3.1 attenuated Aβ-induced indirect neurotoxicity and decreased Aβ-mediated damage to dendrites and synapses by an indirect, astrogliosis-mediated mechanism. In the AD mouse model, levels of pro-inflammatory factors and cytokines were significantly up-regulated and these increases were inhibited by TRAM-34.

The tripartite synapses in the CNS are formed by pre- and post-synaptic neuronal compartments and astroglial perisynaptic processes (Verkhratsky et al., 2010). A phenotype switch of astrocytes may lead to abnormal release of gliotransmitters, such as glutamate and GABA, which could lead to synaptic loss, excitotoxicity, and neurodegeneration in AD (Agulhon et al., 2012; Jo et al., 2014). Numerous clinical studies have confirmed a correlation between the degree of dementia and the extent of synaptic loss. In our studies, blockade of KCa3.1 rescued memory deficits, and synapse/neuron loss in Tg^APP/PS1^ mice. After treatment with TRAM-34, quantification showed that senile plaque formation was decreased in the cortex (**Figure 5B**) but not in the hippocampus (**Figure 5D**). We propose that the different efficacy with respect to different brain areas is that the hippocampus was the greatest amyloid burden region in mice model of AD (Reilly et al., 2003), and the processes of astrogliosis mostly observed surrounding amyloid plaques also were able to accumulate large amounts of senile plaque (Rodriguez et al., 2009). Reducing astrogliosis is a promising strategy for controlling harmful CNS inflammation in neurodegenerative disorders (Khakh and Sofroniew, 2015; Sofroniew, 2015).

In the present *in vivo* study, we found that KCa3.1 was up-regulated in reactive astrocytes of Tg^APP/PS1^ mice and AD patients compared with WT mice and control humans. It has also been reported that KCa3.1 is involved in several aspects of microglial activation *in vitro* (Kaushal et al., 2007; Maezawa et al., 2011; Ferreira and Schlichter, 2013). Maezawa et al. (2011) reported that KCa3.1 activity is required for Aβ-induced microglial neurotoxicity *in vitro*. In our study, KCa3.1 channels were rarely co-localized with microglia, although blockade of KCa3.1 reduced both astrogliosis and microglial activation in Tg^APP/PS1^ mice. Based on these results, we propose that the expression of KCa3.1 is under the immunohistochemically detectable levels in microglia of both mice and human. Co-cultures of astrocytes and neurons were established to determine whether or not KCa3.1 is involved in astrocyte-accelerated Aβ-induced neurotoxicity. Our data suggest that TRAM-34 might reduce Aβ-induced neurotoxicity by suppressing astrogliosis.

The mechanism of the Aβ-induced phenotypic switch of astrocytes and its potential role in the progression of AD provide excellent opportunities for novel therapies such as TRAM-34, a small molecule blocker of KCa3.1. We have provided evidence that KCa3.1 regulates Aβ-induced astrogliosis and neurotoxicity. The anti-inflammatory and neuroprotective properties of KCa3.1 blockers have been demonstrated in many animal models, including traumatic brain injury, retinal ganglion cell degeneration, and multiple sclerosis (Mauler et al., 2004; Reich et al., 2005; Kaushal et al., 2007; Yi et al., 2016b). Our data show that KCa3.1 is also a promising target for reducing inflammatory damage in AD.

## Author Contributions

ZY supervised the entire project, designed research, and wrote the paper. HC conceived and designed the experiments, interpreted and analyzed data, supervised all the experimental procedure. TW and MY conceived and designed the experiments, performed research interpreted and analyzed data. QL performed research and analyzed data. WG and LH analyzed data and critically revised the manuscript.

## Conflict of Interest Statement

The authors declare that the research was conducted in the absence of any commercial or financial relationships that could be construed as a potential conflict of interest.

## Abbreviations

CM-H_2_DCFDA: 5-(and–6)-chloromethyl–2′,7′-dichlorodihydrofluorescein diacetate
GFAP: glial fibrillary acidic protein
KCa3.1: intermediate-conductance calcium-activated potassium channel
TGF-β: transforming growth factor-β
TRAM-34: 1-[(2-chlorophenyl)(diphenyl)methyl]-1H-pyrazole
